# Expected spatial patterns of alien woody plants in South Africa’s protected areas under current scenario of climate change

**DOI:** 10.1038/s41598-020-63830-x

**Published:** 2020-04-27

**Authors:** Bezeng S. Bezeng, Kowiyou Yessoufou, Peter J. Taylor, Solomon G. Tesfamichael

**Affiliations:** 1 Department of Geography, Environmental Management and Energy Studies, University of Johannesburg, APK Campus, Auckland Park, 2006 South Africa; 2 School of Mathematical & Natural Sciences, University of Venda, P. Bag X5050, Thohoyandou, 0950 South Africa

**Keywords:** Ecology, Climate sciences, Environmental sciences

## Abstract

Although protected areas (PAs) are declared to provide sanctuaries for biodiversity, they are increasingly threatened by the synergistic effects of anthropic factors, invasive alien species and climate change. Consequently, interventions are required to minimize the impacts of these threats on PAs’ integrity. To inform these interventions in the South African context and under the current climate change scenario, we tested for geographic patterns of alien woody species across the network of 1,453 PAs using three alien invasion indices – alien species abundance, invaded area ratio and alien species richness. Our analysis shows that, under current climate change scenario, none of the PAs would be effective in shielding against alien plants and PAs that are geographically close tend to share similar invasion patterns. In addition, PAs that are hotspots of alien species are also geographically clustered but these findings are biome-dependent. Our outlier analysis reveals not only an island of disproportionately rich PAs in alien species, but also identifies some alien-poor PAs. We suggest that PAs that are hotspots of alien species as well as outliers of disproportionately rich PAs in alien species should be priority in monitoring and invasion control programmes in the context of the ongoing climate change.

## Introduction

Protected areas (PAs) represent key ecological units for the conservation of native species to ensure the continuous provision of ecosystem goods and services^1–4^. However, mounting evidences now show that invasive alien species threaten several PAs across the globe^5–9^, causing significant loss of biodiversity and jeopardizing the ecological integrity of PAs. For example, a recent global study revealed that invasive alien species, which are ranked fifth on the list of the global drivers of biodiversity loss (see ref. ^10^), can be associated with the loss of up to 58% of amphibians, birds, mammals, plants, and reptiles^11^. As such, if invasive alien species are poorly managed, the future persistence and values of many PAs would be severely impacted^12^. This problem is further compounded by the synergistic interactions between anthropic factors, invasive alien species and climate change^13,14^.

A common approach to assess and monitor alien plants and related invasion characteristics employs the machine learning algorithms that use climate data as inputs [e.g.^15–18^]. This approach is particularly useful in developing early warning systems that inform effective alien plant management^19,20^. A typical example in this regard is Environmental Niche Modelling (ENM), which predicts a species’ past, current, and future areas of suitability by relating environmental parameters with a species’ geographic distribution^15,21–24^. Although this approach provides information that are solely location-related, it has found widespread applications in the field of ecology^15,25,26^. A number of studies have been conducted to map the spatial distribution of plants worldwide. For example, a recent study identified localized hotspots and coldspots of seagrass in eastern Canada^27^. Such analysis demonstrates the simplest form of invasion area mapping by focusing on a single species. Also, similar studies mapped hotspots and coldspots of invasive species diversity and spatial coverage by dividing a part of the USA forested ecosystems into hexagons of 1,452 km^2^ (e.g. ref. ^28^). Although the approach followed by ref. ^28^ is applicable to a large-scale assessment of alien geographic patterns, it did not provide information specific to the invasion pressures that different types of PAs are facing.

Furthermore, despite the growing interest in spatial pattern analysis of vegetation, simultaneous comparisons of invasion patterns across different land cover types and PAs are understudied^29,30^. Of relevance though is the study by ref. ^9^ who compared invasion patterns across major biomes at a global scale. Although their investigation incorporated PAs, it did not capture national-level biome categorization that has more localized details or relevant information for conservation, which might be overlooked in global-scale analysis. In addition, the same study targeted 36 species that are considered the worst global invaders^9^, with again, no clear highlights of how invasion at local scale may inform the management of invasive species in PAs at country-level. More critically, we have limited understanding of how different PA classifications perform in the face of alien invasion particularly in the face of climate change threat. This understanding is necessary since different classifications of PAs are expected to influence invasion patterns^31^.

The present study aims to characterize the expected spatial distribution of alien woody vegetation across all South Africa’s current network of PAs under current climate change. Specifically, we aim to (1) assess the spatial distribution of selected alien invasion indices, and (2) compare spatial distribution patterns across biomes and PA classifications. We focused our analysis on three invasion indices: i) abundance of invaders; referring to the total count of alien species in PAs, ii) ratio of invaded area; quantified as the size of invaded area in a PA divided by total size of the PA, and iii) species richness of invaders; referring to the count of alien species within a PA. Spatial patterns of these indices were analysed using global spatial autocorrelation, hotspot analysis and outlier analysis. The global spatial autocorrelation, which is analyzed using the Global Moran’s I index, shows generic spatial patterns across the study area. The hotspot analysis, expressed using Local Moran’s I index, provides an indication of high and low values of invasion indices at localized spatial scale. The outlier clustering, quantified using Anselin Local Moran’s I index, builds on hotspot analysis by identifying unusual concentration level within a neighbourhood of invasion status (hotspot or coldspot). The study uses more than 1,400 PAs found in South Africa across all biome types of the country. As such, it is the most comprehensive study in the country’s PAs that are affected by many invasive species^15,32,33^.

## Results

### Spatial patterns of invasion indices

Using the Global Moran’s I statistic, our analysis shows that all invasion indices are significantly clustered (Table 1), that is, PAs that are geographically close tend to share similar invasion indices.

**Table 1:** Global Moran’s I and associated results of invasion status indicators.

Invasion indicator	Moran’s I index	Z-score	Distribution pattern
Species abundance	0.055059	8.41***	Clustered
Invaded area ratio	0.129343	19.04***	Clustered
Species richness	0.572429	83.61***	Clustered

***p < 0.01.

**Figure 1:**
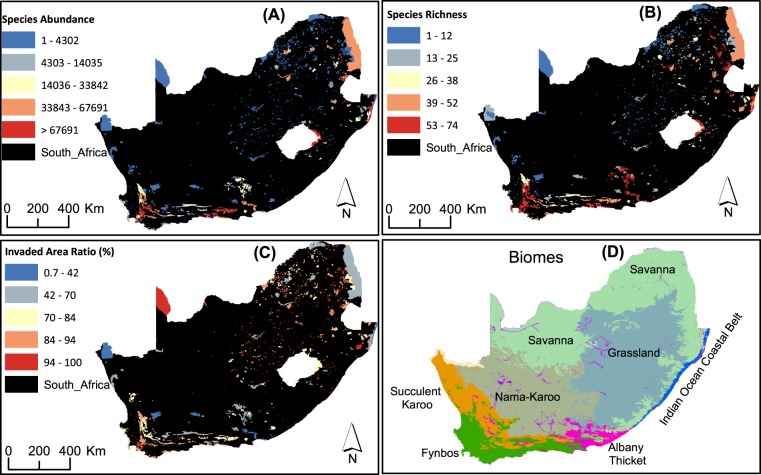
Geographical distributions of the clusters for each invasion index: (**A**) species abundance (**B**) species richness (**C**) invaded area ratio (**D**) geographic pattern of biomes highlighting the most dominant biome types in each PA. Maps were prepared by the authors using ArcGIS software version 10.6.

PAs with high abundance of alien plants (>33,843 total count of alien plants) are clustered in the north-east, eastern and southern parts of the country (Fig. 1A); these areas are located mostly in Savannah, Grassland, Fynbos and Albany Thicket biomes. PAs with lower abundance of alien plants are also clustered but predominantly in the central and western parts of the country, coinciding mainly with the Succulent Karoo and Nama-Karoo biomes. Similar geographic clustering patterns are also observed for species richness (Fig. 1B). However, the spatial pattern of PAs based on invaded area ratio is more widespread than the other two invasion indices (Fig. 1C; see also Fig. 1D relating all three invasion indices to the most dominant biome types in each PA).

**Figure 2:**
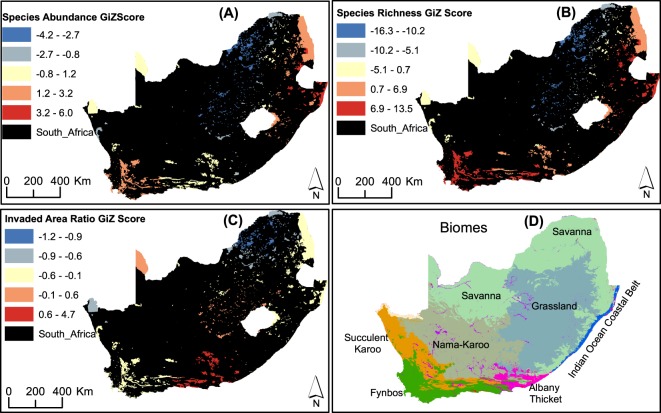
Distribution of protected areas according to clustering extent of (**A**) species abundance **(B**) species richness (**C**) and invaded area ratio High clusters (hotspots) are represented by high positive Gi Z scores while low clusters (cold spots) are represented by low negative Z scores. Z values in between show non-significant clustering patterns. (**D**). Biomes are shown to provide reference to the hotspot and cold spot distributions. Maps were prepared by the authors using ArcGIS software version 10.6.

Furthermore, the distribution analysis taking into account localized neighbourhood patterns was run using the Getis-Ord Gi statistic. This analysis produced hotspots and coldspots of invasion indices (Fig. 2). Hotspots of species abundance are found in the eastern (Savannah and Grassland Biomes) and south-western (Fynbos biome) parts of the country while coldspots of species abundance are distributed in the geographic band stretching from the central to the northern portion of the country within Nama-Karoo and parts of Grassland and Savannah biomes (Fig. 2A). Random distribution patterns are shown mainly in the western and southern parts of the country. A distribution pattern similar to that reported for species abundance was observed for species richness; however, the entire southern part of the country (Fynbos and Albany Thicket biomes) is classified as hotspot of species richness (Fig. 2B). Looking at the invaded area ratio (Fig. 2C), hotspots are found predominantly in the south-eastern part (mainly in Albany Thicket biome) of the country while coldspots are located in the northern part (Savannah biome; see Fig. 2D for easy referencing of the different biome types to the hotspot and coldspot distributions).

A large number of PAs are located within hotspot (n = 492) and coldspot (n = 556) of abundance of alien species (Table 2). More than half of PAs are classified as coldspots or hotspots of invaded area ratio; in contrast, the distribution of invaded area ratio appears to be random in approximately 3% of PAs. However, with species richness of alien species, more than 96% of PAs belong to hotspot or coldspot categories (Table 2).

**Table 2:** Number of protected areas categorized as hotspots, coldspots and randomly distributed in terms of invasion indices.

Status	Species abundance		Invaded area ratio		Species richness	
	GiZ*	Number of PAs	GiZ*	Number of PAs	GiZ*	Number of PAs
Hotspots	≥1.65	492 (33.9%)	≥1.71	711 (48.9%)	≥1.64	468 (32.2%)
Coldspots	≤−1.65	556 (38.3%)	≤−1.65	701 (48.2%)	≤−1.65	276 (19%)
Random*	−1.65–1.65	405 (27.9%)	−1.56–1.71	41 (2.8%)	−1.65–1.64	709 (48.8%)

^*^Z scores with p < 0.1.

**Figure 3:**
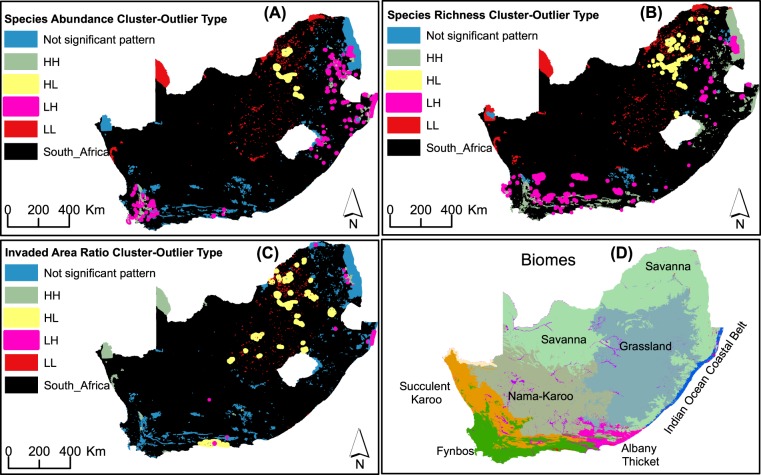
Distribution of protected areas detecting outliers within clusters (neighbourhoods) of high and low values of (**A**) species abundance (**B**) species richness and (**C**) invaded area ratio. HH = high value within a cluster of High values; HL = High within Low cluster; LH = Low within High cluster; LL = Low within Low cluster. (**D**) Biomes are shown to provide reference to the distributions of outliers. Maps were prepared by the authors using ArcGIS software version 10.6.

Finally, the distribution of outliers-within-clusters of invasion indices assessed using the Anselin Local Moran’s I statistic is shown in Fig. 3. This analysis reveals different geographic scenarios. First, there are regions that have no outliers of PAs, that is, PAs in these regions share similar invasion indices. This is the case for 108 PAs in the far south-western part and in the eastern parts of the country where alien abundance is equally high in all 108 PAs (HH category of PAs; Supplementary Table S1). Second, some PAs can be regarded as islands (n = 9) in their regions as they harbour unusually higher alien abundance than their neighbouring PAs (HL category); these are found towards the central to northern part of the country (Supplementary Table S1). A significant number of PAs are identified as outliers of high species richness and invaded area ratio. However, they are both concentrated in the northern part. In general, PAs in HL category are found predominately in the Savannah biomes for all indices. The low abundance outliers within the high abundance clusters (LH category) occur in the eastern and far south-western parts (Fig. 3A) with a total number of 142 PAs (Supplementary Table S1). Similar patterns of LH distribution were observed for species richness; however, the outliers in the south-west is comprised of more PAs than in the east (Fig. 3B). Outliers in terms of invaded area ratio were more widespread than those of the other indices, stretching from the central to the north-eastern part of the country (Fig. 3C). Similar to HL distributions, a generic pattern was observed for LH category for the three indices mostly in the Grassland and Fynbos biomes (Fig. 3; see Fig. 3D relating the different biome types for easy referencing to the distribution of outliers).

### Invasion comparison by biome type

PAs in Forest biome contain the highest mean number of alien woody species while PAs within the Succulent Karoo biome have the lowest mean number of invaders (Supplementary Table S2). The invaded area ratio appears to be comparable across biomes with the mean ranging between 86 (Azonal Vegetation) and 96 (Succulent Karoo). A considerable variation is observed among biomes for species richness of alien plants, with an average of 46 species recorded in PAs found in Forest biome. By comparison, Nama-Karoo and Succulent Karoo biomes have the lowest mean species richness (Supplementary Table S2).

**Figure 4:**
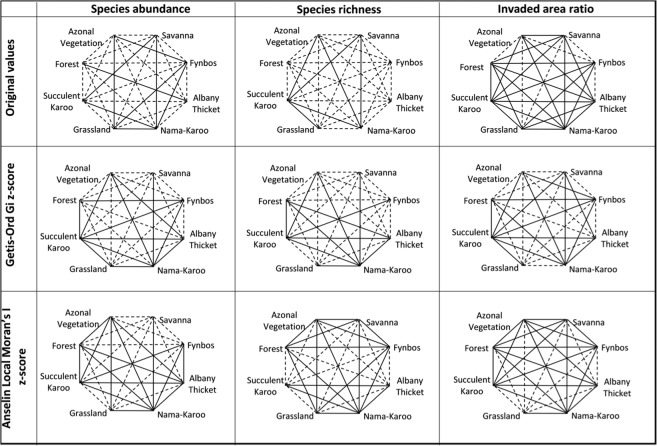
Pairwise comparison of invasion indices by biome type using original values, Getis-Ord Gi z-score (hotspot/cold spot) and Anselin Local Moran’s I z-score (cluster–outlier statistic). Dashed lines indicate significant difference and solid lines indicate non-significant difference. Significance is measured at p = 0.05.

In term of pairwise comparison across biomes, 19 pairs out of the 28 possible pairwise comparisons, showed significant differences in terms of the original values of species abundance (Fig. 4). For example, there was significant difference between the Forest biome and all the other biomes, except the Albany Thicket biome. Similarly, the pattern of alien species richness was significantly different from most of the remaining biomes (Fig. 4). As for the invaded area ratio, its pattern did not vary significantly among most biome types, except Azonal Vegetation vs. Fynbos (Fig. 4). Similarities in the three invasion indices were observed consistently between Nama-Karoo vs. Succulent biomes, Savannah vs. Grassland as well as Azonal Vegetation vs. Forest biomes.

In addition, the comparison of the spatial distribution pattern of species abundance using hotspot statistic (Getis-Ord Gi) showed significant differences among PAs, depending on the dominant biomes in these PAs (Fig. 4). For instance, species abundance varied significantly between the Savannah biome and all the other biomes, except the Nama-Karoo. Forest and Grassland had significant differences in terms of species abundance with all other biomes, except for two biomes. In contrast, the Nama-Karoo and Succulent Karoo had non-significant differences with six other biomes. The results for invaded area ratio and species richness comparisons were generally similar to those obtained for species abundance.

The pairwise comparisons of biomes based on outlier statistics (Anselin Local Moran’s I statistic) showed significant differences among biomes (Fig. 4). In particular, the Savannah biome varied significantly from all other biomes except the Nama-Karoo, whilst Grassland had significant difference with six other biomes. In contrast, the Nama-Karoo, Azonal Vegetation, Albany Thicket, Forest and Succulent Karoo had comparable outlier-cluster patterns of species abundance with six other biomes. Compared to species abundance, few significant differences were observed for outlier-cluster pattern of invaded area ratio. Nearly all the significant differences in invaded area ratio were observed between Grassland and the rest of biomes as well as between Fynbos and Albany Thicket. The outlier-clustering patterns of species richness varied significantly among a number of biomes. The Succulent Karoo and Albany Thicket had significant differences with five other biomes, while the Nama-Karoo had an outlier-cluster pattern comparable with all the other biomes, followed by Azonal Vegetation, Forest and Fynbos with each showing similarity with five biomes.

### Invasion comparison by PA classifications

The grouping of PAs by class shows that PAs in Mountain Catchment Area has the highest mean abundant value of alien plants (n = 36,373; Supplementary Table S2). Unlike the per-biome observations, there were more variations in the mean invaded area ratio of PAs among classifications with Forest Nature Reserve, Nature Reserve and Special Nature Reserve having the highest mean invaded area ratio. However, the statistics for Special Nature Reserve is based on only one PA and thus may not be considered conclusive. PAs in Mountain Catchment Area, Forest Nature Reserve and Forest Wilderness Area have the highest mean number of alien woody species (n ≥ 40).

**Figure 5:**
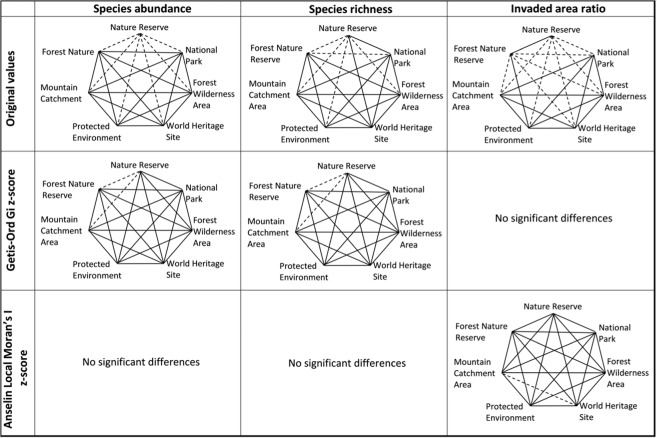
Pairwise comparison of invasion indices by PA classifications using original values, Getis-Ord Gi z-score (hotspot/cold spot) and Anselin Local Moran’s I z-score (cluster–outlier statistic). Dashed lines indicate significant difference and solid lines indicate non-significant difference. Significance is measured at p = 0.05. Note that Getis-Ord Gi statistic for invaded area ratio as well as Anselin Local Moran’s I statistic for species abundance and species richness did not vary among management regimes; therefore the graphs are not included in the figure.

The comparisons between PA classifications using the original invasion indices were relatively similar for the three invasion indices (Fig. 5). Notably, significant differences in species abundance were observed between PAs under Nature Reserve vs. each of the other classes. In comparison to species abundance, most of PA types showed significant difference in terms of invaded area ratio. For instance, invaded area ratio of PAs in Forest Nature Reserve varied significantly from those in four other PAs. Significant differences in species richness between PA types were largely similar to those observed for species abundance (Fig. 5).

There were no significant differences in the hotspot/coldspot patterns (Getis-Ord Gi z-score statistic) for invaded area ratio among PAs (Fig. 5). Comparisons of the patterns among PAs were similar for species abundance and species richness. In both cases, the only significant differences were observed between PAs in Nature Reserve vs. Forest Nature Reserve as well as Nature Reserve vs. Mountain Catchment Area. The cluster-outlier distribution pattern (Anselin Local Moran’s I z-score statistic) returned non-significant differences among all PA types for species abundance and species richness. Only one significant difference was observed between PAs in Mountain Catchment Area vs. World Heritage Site for invaded area ratio.

## Discussion

Our global distributional analysis showed unambiguously some clustering patterns of PAs with respect to the three invasion indices used. These clustering patterns of PAs using species abundance and species richness were very similar, suggesting that abundance of alien plants predicts their richness in geographically close PAs in South Africa. These patterns could be caused by different factors such as relatively easy dispersal of alien plants in shared environmental conditions (soil, water, climate, biomes types, etc.) among PAs in the same geographic regions^25^. However, for the index “invaded area ratio”, the clustering pattern of PAs is less similar to the patterns found for species abundance and species richness. This is due to the limited variation in “invaded area ratio” values across biomes (coefficient of variation 15%) compared to the 75% and 406% for species richness and species abundance, respectively (Supplementary Table S3).

Furthermore, localized hotspot/coldspot results show strong similarities in the distribution of species abundance and species richness, as was observed for the global cluster distributions. The other noticeable similarity was the relatively equal number of PAs categorized as hotspots and coldspots of species abundance and species richness (Table 2). The large number of hotspots of species abundance and species richness is a concern as this suggests that most neighbouring PAs are highly invaded; this calls for integrated management efforts of alien invasive plants in PAs. Similar to the observation in global clustering, the local hotspot and coldspot distributions of abundance and species richness did not coincide with those of invaded area ratio. There is, however, an interesting pattern of the hotspot and coldspot distributions of invaded area ratio. All spatial pattern outcomes (hotspot, coldspot, random) of invaded area ratio stretch contiguously over large spatial areas, compared to the more localized variations observed in the other two indices (Fig. 2).

Although PAs are clustered irrespective of the index used, some might be unusually high or low in alien species in the same geographic region. To explore this possibility, cluster-outlier analysis is relevant in the context of invasive species mapping, since it identifies an unusual invasion pattern within a neighbourhood of PAs. We found clear patterns of both HL (high values of invasion indices within clusters of low values) and LH distributions. The HL distributions are predominantly related to the Savannah biome generally characterized by grasses interspersed to a varying degree by woody vegetation. As such, a PA with alien woody vegetation would easily stand out in a cluster of grass-dominated PAs. Similarly, the LH distributions for the three invasion indices can be linked to the biome types they are mostly located in (Fig. 3). For example, the Fynbos biome consists of a relatively complex plant composition ranging from the simple plant lifeforms to woody vegetation types; it is therefore expected to find a low invasion of woody alien plants within a cluster of high invasion of woody vegetation. The LH association with Grassland biome firstly needs to be traced back to the fact that woody vegetation encroachment is a common problem threatening the biome, e.g. see ref. ^34^. However, it is logical to expect patches of Grasslands with limited invasion of woody vegetation surrounded by those with high invasion status. Finally, it is important to note the results showing more LH than HL occurrences for all the invasion indices (Supplementary Table S1). This can be expected given that the goal of PAs is to maintain the natural (indigenous) vegetation complexity; therefore, more LH than HL is perhaps indicating that management efforts of PAs are contributing effectively to safeguarding PAs against invaders. However, our finding of a variation in invasion intensity across different biomes and ultimately different PAs is not a surprise given that a recent study showed that biome types constrain, to different degrees, the distribution of alien species assemblages in South Africa^35^. Indeed, confirming the findings of ref. ^35^, we found that invasion status was generally associated with biome and PA types as evidenced by significant differences in the original and derived statistics of invasion indices (Fig. 4; see also refs. ^35,36^). This significant difference is not a surprise. For example, the difference between Forest biome (which has the highest mean species abundance and species richness; Supplementary Table S2) and all the others, except the Albany Thicket biome, is related to the fact that forest ecosystems are more likely to provide suitable ecological niches for alien woody plants than other non-woody ecosystems^35^.

It is also important to note the consistent significant difference between Grassland and Savannah biomes in terms of vulnerability to alien woody plants invasion. The higher invasion status of Grassland than Savannah is surprising, given that Savannah naturally hosts some woody species, and consequently we should expect that Savannah would be more vulnerable to alien woody invasion than Grassland. Our opposite finding is indicative of the significant bush encroachment in the Grassland, and this encroachment will eventually convert current Grassland into Savannah if the encroachment continues^34^. This potential decrease of Grassland biome (partly due to woody encroachment) was also predicted in an early study where climate change has been pointed out as a driving force of shrinkage of the Grassland biome in South Africa^37^. The encroachment of alien woody plants in Grasslands also means that ecological niches for woody plants may be available in Grasslands, and alien woody plants are simply taking advantage of this niche availability favoured by weak competitive ability of grasses *vis-à-vis* woody plants Darwin naturalization hypothesis^38,39^. Nevertheless, the lack of significant differences between Forest and Albany Thicket biomes (irrespective of the invasion index used) indicates that both biomes are equally vulnerable to the invasion by woody alien plants. This is not a surprise because Albany Thicket is naturally dominated by woody bushes and shrubs^40^, and thus its vulnerability to the invasion by alien woody plants is comparable to that of the Forest biome. Another notable comparison of invasion by biome type was the non-significant difference between the Nama-Karoo and Succulent Karoo biomes in all comparisons (Fig. 5). The Nama-Karoo and Succulent Karoo are characterized by limited precipitation and high temperature conditions that do not favour the proliferation of woody vegetation; as a result, invasion status in the two biomes is low (Supplementary Table S2). By extension, the comparability between the two biomes is expected, as found in this study, to hold for hotspot/coldspot as well as cluster-outlier patterns.

What’s more, studies assessing the relationship between invasion and different PA types are rare. One of the few existing studies (e.g.^41^) showed a non-significant difference in number of invasive and managed species between national parks and biosphere reserves, although they used a far less number of PAs than used in our study. In our study, comparison of invasion indices among PA types returned non-significant differences in most instances (Fig. 5). One notable consistency was the significant difference of invasion between Nature Reserve and the other PA types. This classification type (Nature Reserve) did not have the highest invasion status in any of the three indices. It is meaningful to evaluate the invasion comparisons of PA types against the expected management level, as this would assist in determining if PAs are achieving their stated goals.

The results presented in this study have important implications for PAs management in South Africa and perhaps elsewhere. This is partly because several countries in the world including South Africa have seen a rise in new PAs declaration due to renewed commitments through national biodiversity strategies and action plans (NBSAP) to protect species and habitats. As such, much attention has been given to PAs because, in addition to serving as refugia for a diversity of species, they play an important role in mitigating the effects of climate change^42^. As a result, the IUCN’s World Commission on Protected Areas (IUCN WCPA) has championed the development of methodologies to assess PAs’ management effectiveness (PAME^43^). Despite this, many PAs are still highly threatened by human activities^9,44^, while changing climatic conditions may exacerbate the extent of alien invasion across PAs [e.g.^45^]. The findings of the present study regarding the spatial distributional patterns of selected invasion indices (as predicted under current climate change) provides useful information that can be used to support management strategies. Indeed, the high spatial clustering of PAs based on alien woody invasion indices in some parts of the country is an indicator of where the prioritization of management and monitoring efforts should be focused. Focussing on these areas is further justified by the fact that they are also identified as hotspots of invasion (see also ref. ^15^).

The findings of this study also showed similarities between species abundance and species richness, when using invasion indices of the original values and those interpreted from hotspot and coldspot distributions. This indicates the co-success of both invasion characteristics (abundance and richness), although this can be curtailed when the PAs are “saturated”. An important lesson from this finding is that, management strategies should exercise caution not to assume that intra- and inter-species competitions among alien species in South Africa’s PAs may help to reduce their species richness. In fact, based on our findings, it is safe to expect high species richness when there is high abundance of alien plants, at least in the case of South African PAs. This expectation may also translate into management strategies in that efforts to reduce species abundance may result in reducing species richness too. In the South African context, physical removals of alien plants are a well-known management strategy of alien species. Our results suggest that, by physically removing alien plants, we are not only reducing alien abundance but also alien species richness. However, we should also note that the effectiveness of this physical removal of alien is not a guarantee of success because the seed bank of alien plants in the soil as well as their rhizomes are not affected by physical removals. Some have suggested the use of natural enemies or chemicals to control alien invasive species but we vigorously oppose these solutions in PAs. We advocate for the practice of periodic physical removals of alien plants in PAs^46^, assuming that the constant reduction of seedlings, juveniles and adults of alien plants would eventually lead to a decrease of the population dynamics of these alien species and the increase in native plants. Our suggestion is supported by a recent study that demonstrated that physical removals of all alien invasive plants leads to an increase of native plants and pollinators^46^.

Furthermore, our cluster-outlier analysis revealed that, in some geographic regions, some PAs are disproportionately richer in alien plants in comparison to their respective neighbouring PAs (HL), and other regions harbour disproportionately poorer PAs in alien plants than their neighbouring PAs (LH). These findings have clear management implications. Disproportionately-rich PAs in alien plants should be the focus of intensive physical removals of alien plants^46^ as they represent a serious threat not only to their own native biodiversity and habitats but also to the neighbouring PAs. They are a threat because not only they most likely share similar environmental characteristics with neighbouring PAs, but also geographic proximity is a conducive factor for rapid dispersal of alien plants through wind^47^ or through biotic mediation, e.g. mutualist interactions^48^. At the same time, PAs that are poorer in alien plants but surrounded by richer PAs in alien plants should also be under particular surveillance and monitoring such that early detection programmes of alien plants should be designed for these PAs while alien-rich PAs should undergo an intensive physical removal programme.

We acknowledge some limitations of the present study. For example (i) we focused only on PAs that are spatially discontinuous as opposed to contiguous landscapes that consist of different land uses and (ii) the sample size of PAs was unbalanced with Nature Reserve in particular being the dominant PAs type in our dataset. These limitations were an unavoidable data characteristic, and we believe that the choice of our data analysis such as comparison of invasion indices using the Kruskal-Wallis^49^ statistic is deemed fit to counter these concerns. Furthermore, it is important to note that this study does not attempt to determine the effect of biome and PA classifications on invasion characteristics. Lastly, the invasion indices used were calculated based on predictions under current climate change scenario.

## Conclusion

To conclude, we encourage further studies that can better the limitations of this study; e.g. studies geared towards understanding the potential drivers of invasion status or patterns using biomes and PA classifications as explanatory variables. Such analysis will therefore provide more than the simple analysis of variance (association) adopted in this study. Satisfactory prediction potential based on biomes and PA classifications will go a long way in designing biome-specific or management-specific of PAs in the context of alien invasion. Additionally, this study focused on non-native woody vegetation only; it is vital to expand such studies to encompass other lifeforms as more data become available. Finally, there is a need to incorporate remote sensing in plant invasion management, since the technique provides an efficient and unbiased method of plant species and habitat dynamics monitoring at multiple scales^50^.

## Material and Methods

### Current climate distribution of alien woody species

Data on the distribution of alien woody species were retrieved from^15^. In summary, using ecological niche modelling (ENM) approach, ref. ^15^ reconstructed species richness maps for 162 alien woody trees and shrubs in South Africa based on 19 bioclimatic variables (Supplementary Table S4). These maps were calibrated using >87,000 point occurrence data as well as current climate data at a spatial resolution of ~1 km^2^. These occurrence data are publicly available on Dryad (https://doi.org/10.5061/dryad.4j0zpc87q)^51^. To ensure robustness of the species richness map, they combined two approaches. First, MaxEnt method [i.e. a presence only data^52^] was used as it works well with species that have fewer occurrence points (i.e. a minimum of 8 occurrence points in their case). Second, they also used an ensemble forecasts method (i.e. presence-absence data) comprising three algorithms: generalized linear models, random forests, and gradient boosting machine^53–56^. The resulting species richness map had a grid resolution representing 1,000 m on the ground, in agreement with the spatial resolution of the climate data that served as inputs in the modelling. In the present study, the grid data were converted to polygons on which PAs and biomes distribution were overlaid.

### Network of Protected Areas and biome data in South Africa

We obtained spatial data on PAs from South Africa Protected Areas Database (SAPAD) of the Department of Environmental Affairs (http://egis.environment.gov.za). The database maintains the status of eight formal and less formal PAs on a quarterly basis. We used the latest release representing the second quarter of 2018 (see Fig. S1). A quick comparison of the releases since the second quarter of 2017 did not show significant differences that would change the results of our analyses. In total, 1,453 PAs are included in the present analysis, including state- and privately-owned PAs. A systematic management standard governing South Africa’s protected areas is recognized in the National Environmental Management: Protected Areas Act of 2003. The most dominant biome types and PA classifications were also identified (Table 3; Supplementary Fig. S1).

**Table 3:** Number of protected areas (PAs) corresponding to each biome and PA classifications recognized in this study. Note that only one PA is classified as Special Nature Reserve; as a result, this PA was not used in statistical analysis comparing invasion status across PA classifications .

Biome types	Number of PAs	Area (ha)	PA Classifications	Number of PAs	Area (ha)
1. Albany Thicket	77	1071749	1. Forest Nature Reserve	49	169968
2. Azonal Vegetation	320	7026587	2. Forest Wilderness Area	12	274490
3. Forests	117	701462	3. Mountain Catchment Area	16	624567
4. Fynbos	171	315140	4. National Park	21	3978614
5. Grassland	287	883925	5. Nature Reserve	1310	3843717
6. Nama-Karoo	7	21035	6. Protected Environment	24	593216
7. Savannah	467	1471076	7. Special Nature Reserve	1	2
8. Succulent Karoo	7	17800	8. World Heritage Site	20	2024199

Also, a spatial coverage showing the biome types and hierarchical sub-divisions of biomes of South Africa was downloaded from the South African National Biodiversity Institute’s (SANBI) website (http://pza.sanbi.org/vegetation). The data represents the latest available information updated in 2012. Floristic characteristics such as dominant life or growth forms and, to a lesser extent, macroclimatic characteristics were used to define biome types in South Africa^57^.

### Invasion status indices of PAs

We used three indices to quantify the invasion status of all 1,453 PAs: abundance of invaders (alien woody plants), ratio of invaded area and species richness of invaders. Abundance of invaders was estimated as follows: we first employed the ENM technique to get the species richness map for the whole country based on the 19 bioclimatic data. Secondly, we overlaid the network of PAs onto the species richness map. Finally, we extract the number of species found exclusively within the boundary of PAs. For a PA, the ratio of invaded area was computed as the area covered by all alien species divided by the total area of the PA. Species richness of invaders simply corresponds to the total count of unique alien species within each PA (see Supplementary Table S3 for the summary statistics of all three metrics).

### Statistical analyses

#### Spatial pattern analysis of alien species

Spatial analysis was carried out to test the spatial pattern (random vs. dispersion vs. clustering) of alien species in PAs based on the invasion status indices (ratio of invaded area, species richness of invaders and abundance of invaders). Specifically, we used a global spatial autocorrelation and two local spatial pattern indicators including hotspot analysis and cluster–outlier analysis. Spatial autocorrelation was tested using the Moran’s I index^58^ defined in Eq. 1. This statistic assumes that spatial processes are consistent across the study area. We applied this specific statistic since there was no prior knowledge in South Africa of whether spatial patterns of plant invasions are localized or more generic. As such, applying this statistic in our study could provide an indication of whether the assumption of ‘global’ consistency holds. According to the Moran’s index, a spatial pattern (autocorrelation) is said to exist between features if a feature’s value deviates from the global mean value significantly.

1 $$I=\frac{N}{W}\frac{{\sum }_{i}{\sum }_{j}{w}_{ij}({x}_{i}-\bar{x})({x}_{j}-\bar{x})}{{\sum }_{i}{({x}_{i}-\bar{x})}^{2}}$$

where *I* is Moran’s *I*, *N* is number of protected areas used in the analysis; *x*_*i*_ and *x*_*j*_ are the values of the invasion indices defined above (e.g., species richness) for two protected areas whose spatial separation is being quantified; $\bar{x}$ is the mean value of interest of all protected areas, *w*_*ij*_ is the weight of separation distance between two protected areas with a weight inversely proportional to separation distance, and *W* is the sum of all weights. The Moran’s *I* value customarily is transformed to a standard z-score which computes variable of interest (e.g. species richness) of a PA relative to the overall mean and standard deviation values of a variable of all PAs. This transformation allows for testing the hypothesis of whether or not there is spatial autocorrelation in the variable.

In addition to Moran’s *I* autocorrelation, we also performed hotspot analysis to compare the invasion status of a PA against the status of neighbouring PAs^59^. The hotspot was quantified using the Getis-Ord Gi* statistic (Eq. 2). Similar to Moran’s *I* spatial autocorrelation, ${{\rm{G}}}_{i}^{\ast }$ is transformed to z-score to determine whether or not the distribution satisfies the hypothesis that there is clustering of protected areas with high and low variable of interest (e.g., species richness). A statistically high positive z-score indicates an intense clustering of high values known as hotspots, while a statistically negative low z-score shows an intense clustering of low values (coldspots). However, high values within a cluster of high values (HH) or low values within a cluster of low values (LL) are indicative of the absence of an outlier within a neighbourhood.

2 $${{\rm{G}}}_{i}^{\ast }=\frac{{\sum }_{j=1}^{N}{w}_{i,j}{x}_{j}-\bar{x}{\sum }_{j=1}^{N}{w}_{i,j}}{s\,\sqrt{\frac{[N[{\sum }_{j=1}^{N}{w}_{i,j}^{2}-{({\sum }_{j=1}^{N}{w}_{i,j})}^{2}]]}{N-1}}}$$

where ${{\rm{G}}}_{i}^{\ast }$ is the Local G-statistic (Getis-Ord), s is the standard deviation of the variable.

The ${{\rm{G}}}_{i}^{\ast }$ statistic uses each spatial feature when quantifying the cluster intensity of a neighbourhood; as a result, it tends to homogenise a variable per cluster by concealing isolated instances of variation that may exist within clusters. Such isolated occurrences can be identified using cluster–outlier analysis. This cluster–outlier analysis was done in this study using Anselin Local Moran’s I statistic (Eq. 3) which implements the computation in two sequential steps. The first step calculates clusters of high and low values similar to hotspot analysis computed using ${{\rm{G}}}_{i}^{\ast }$ statistic, but with the target protected area not included in the calculation. The second step compares the value of interest (e.g. species richness) of the target feature against the cluster value, and determines if the target has a value that is considered an outlier to the cluster.

3 $${I}_{i}=\frac{{x}_{i}-\bar{x}\,}{{s}_{i}^{2}}\mathop{\sum }\limits_{j=1,\,j\ne i}^{N}{w}_{i,j}({x}_{j}-\bar{x})$$

A positive *I*_*i*_ indicates the value of a protected area (target) is similar to the value of a cluster it is located in, while a negative value shows the protected area is an outlier of its surrounding cluster. Like the Getis-Ord and the global Moran’s *I* statistics, Anselin Local Moran’s *I* statistics is standardized by converting it to a z-score that is used to test whether or not a target (a protected area) has a variable of interest (e.g. species richness) that varies significantly from the surrounding cluster. All spatial pattern analyses were executed in ArcGIS version 10.6 (ESRI, Redlands, CA).

#### Assessing invasion status by biome types and PA classifications

Analysis of variance was used to assess whether or not invasion status varied by biome types and PA classifications. All the three invasion status indicators (species abundance, invaded area ratio and species richness) were used in the analysis of variance. Two statistics were used for each invasion indicator; these included values of the original variable of invasion indices and the spatial pattern statistics (quantified by Getis-Ord Gi and Anselin Local Moran’s *I*). A histogram-based observation of the data particularly those with few protected areas per biome and PAs classification type showed distribution of values that deviated from the normal. As a result, a non-parametric approach namely Kruskal-Wallis^49^ test was applied to compare the statistics. Kruskal-Wallis is a rank-based statistic that tests if an independent variable of two or more samples or groups of samples differ significantly.

## Supplementary information

Supplementary Figure S1.

Supplementary Table S1.

Supplementary Table S2.

Supplementary Table S3.

Supplementary Table S4.

Supplementary information.

## Data Availability

The datasets generated for this study are publicly available on Dryad (10.5061/dryad.4j0zpc87q).
