# Supplementary figures and images for: Expected spatial patterns of alien woody plants in South Africa’s protected areas under current scenario of climate change
Source: Sci Rep. 2020 Apr 27;10:7038. doi: 10.1038/s41598-020-63830-x (PMC7184613; doi:10.1038/s41598-020-63830-x)

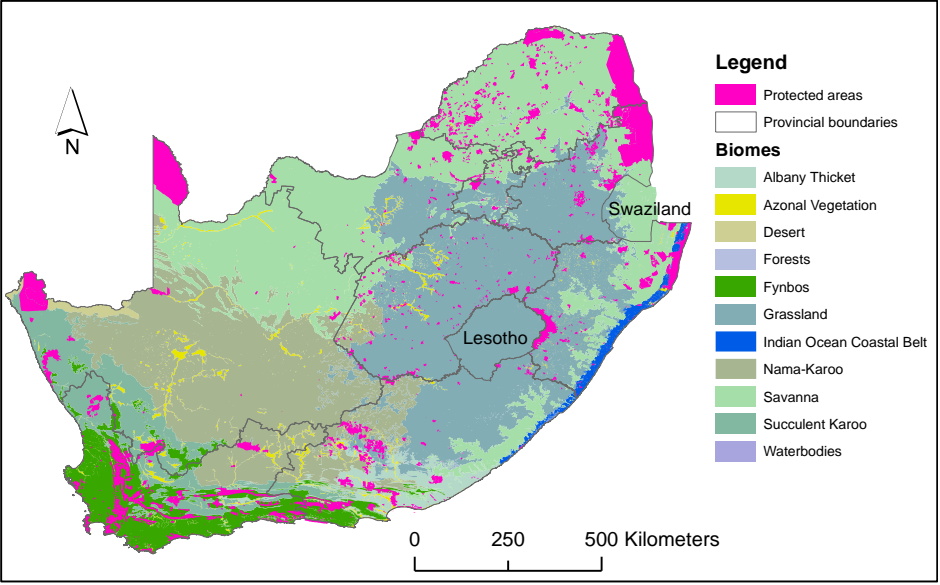

Supplement: Supplementary file 1 — Supplementary Figure S1. [file 41598_2020_63830_MOESM1_ESM.pdf]
