# Supplementary material for: Expected spatial patterns of alien woody plants in South Africa’s protected areas under current scenario of climate change
Source: Sci Rep. 2020 Apr 27;10:7038. doi: 10.1038/s41598-020-63830-x (PMC7184613; doi:10.1038/s41598-020-63830-x)
Supplement: Supplementary file 2 — Supplementary Table S1. [file 41598_2020_63830_MOESM2_ESM.docx]

**Expected spatial patterns of alien woody plants in South Africa’s protected areas under current scenario of climate change**

Bezeng S. Bezeng^1,2*^, Kowiyou Yessoufou^1^, Peter J. Taylor^2^, Solomon G. Tesfamichael^1^

*^1^Department of Geography, Environmental Management and Energy Studies, University of Johannesburg, APK Campus, Auckland Park 2006, South Africa.*

*^2^School of Mathematical & Natural Sciences, University of Venda, P. Bag X5050, Thohoyandou 0950, South Africa.*

Supplementary Table S1: Count of outliers within clusters of high or low invasion indices measured using Anselin Local Moran’s I z-score. HH = high value within a cluster of high values. HH = high value within a cluster of High values; HL = High within Low cluster; LH = Low within High cluster; LL = Low within Low cluster.

|  | Species abundance | Invaded area ratio | Species richness |
| --- | --- | --- | --- |
| Status | Number of PAs | Number of PAs | Number of PAs |
| HH | 108 (7.4%) | 425 (29.2%) | 512 (35.2%) |
| HL | 9 (0.6%) | 71 (4.9%) | 55 (3.8%) |
| LH | 142 (9.8%) | 98 (6.7%) | 94 (6.5%) |
| LL | 595 (40.9%) | 149 (10.3%) | 578 (39.8%) |
| Random* | 599 (41.2%) | 710 (48.9%) | 214 (14.7%) |

*Z-scores with p > 0.1.
