# Supplementary material for: Expected spatial patterns of alien woody plants in South Africa’s protected areas under current scenario of climate change
Source: Sci Rep. 2020 Apr 27;10:7038. doi: 10.1038/s41598-020-63830-x (PMC7184613; doi:10.1038/s41598-020-63830-x)
Supplement: Supplementary file 3 — Supplementary Table S2. [file 41598_2020_63830_MOESM3_ESM.docx]

**Expected spatial patterns of alien woody plants in South Africa’s protected areas under current scenario of climate change**

Bezeng S. Bezeng^1,2*^, Kowiyou Yessoufou^1^, Peter J. Taylor^2^, Solomon G. Tesfamichael^1^

*^1^Department of Geography, Environmental Management and Energy Studies, University of Johannesburg, APK Campus, Auckland Park 2006, South Africa.*

*^2^School of Mathematical & Natural Sciences, University of Venda, P. Bag X5050, Thohoyandou 0950, South Africa.*

Supplementary Table S2: Plant invasion status of protected areas summarized by biome and classification types

|  |  | **Invasion abundance** | | | **Invaded area ratio (%)** | | | **Species richness** | | |
| --- | --- | --- | --- | --- | --- | --- | --- | --- | --- | --- |
| **Biome types** | | | | | | | | | | |
|  | **Count** | **Max** | **Mean** | **Min** | **Max** | **Mean** | **Min** | **Max** | **Mean** | **Min** |
| Albany Thicket | 77 | 114121 | 4771 | 14 | 100 | 87 | 28 | 61 | 31 | 6 |
| Azonal Vegetation | 320 | 212117 | 5529 | 1 | 100 | 85 | 1 | 73 | 25 | 1 |
| Forests | 117 | 169401 | 13655 | 4 | 100 | 92 | 69 | 74 | 46 | 1 |
| Fynbos | 171 | 36417 | 1176 | 2 | 100 | 92 | 7 | 67 | 35 | 2 |
| Grassland | 287 | 67691 | 2066 | 3 | 100 | 92 | 41 | 64 | 23 | 3 |
| Nama-Karoo | 7 | 247 | 56 | 1 | 100 | 93 | 66 | 9 | 6 | 1 |
| Savannah | 467 | 24271 | 583 | 1 | 100 | 89 | 10 | 58 | 14 | 1 |
| Succulent Karoo | 7 | 94 | 27 | 1 | 100 | 96 | 86 | 15 | 9 | 1 |
| **PAs classification types** | | | | | | | | | | |
|  | **Count** | **Max** | **Mean** | **Min** | **Max** | **Mean** | **Min** | **Max** | **Mean** | **Min** |
| Forest Nature Reserve | 49 | 28417 | 6200 | 18 | 100 | 90 | 28 | 67 | 44 | 9 |
| Forest Wilderness Area | 12 | 65266 | 24102 | 2350 | 90 | 81 | 61 | 67 | 40 | 30 |
| Mountain Catchment Area | 16 | 113201 | 36373 | 479 | 93 | 80 | 67 | 71 | 45 | 14 |
| National Park | 21 | 129433 | 16990 | 10 | 100 | 68 | 24 | 73 | 27 | 1 |
| Nature Reserve | 1310 | 169401 | 1623 | 1 | 100 | 90 | 1 | 74 | 23 | 1 |
| Protected Environment | 24 | 56092 | 10305 | 20 | 100 | 84 | 60 | 67 | 37 | 3 |
| Special Nature Reserve | 1 | 3 | 3 | 3 | 100 | 100 | 100 | 3 | 3 | 3 |
| World Heritage Site | 20 | 212117 | 44765 | 4 | 93 | 68 | 36 | 73 | 37 | 2 |
