# Supplementary material for: Expected spatial patterns of alien woody plants in South Africa’s protected areas under current scenario of climate change
Source: Sci Rep. 2020 Apr 27;10:7038. doi: 10.1038/s41598-020-63830-x (PMC7184613; doi:10.1038/s41598-020-63830-x)
Supplement: Supplementary file 4 — Supplementary Table S3. [file 41598_2020_63830_MOESM4_ESM.docx]

**Expected spatial patterns of alien woody plants in South Africa’s protected areas under current scenario of climate change**

Bezeng S. Bezeng^1,2*^, Kowiyou Yessoufou^1^, Peter J. Taylor^2^, Solomon G. Tesfamichael^1^

*^1^Department of Geography, Environmental Management and Energy Studies, University of Johannesburg, APK Campus, Auckland Park 2006, South Africa.*

*^2^School of Mathematical & Natural Sciences, University of Venda, P. Bag X5050, Thohoyandou 0950, South Africa.*

Supplementary Table S3: Summary of invasion status indices of protected areas in South Africa

|  | Invasion abundance | Invaded area ratio (%) | Invasive species richness |
| --- | --- | --- | --- |
| Minimum | 1 | 1 | 1 |
| Maximum | 212117 | 100 | 78 |
| Mean | 3307 | 89 | 24 |
| Standard deviation | 13439 | 13 | 18 |
| 1^st^ quartile | 41 | 86 | 9 |
| 2^nd^ quartile | 189 | 93 | 21 |
| 3^rd^ quartile | 1087 | 98 | 38 |
| Coefficient of variation (%) | 406 | 15 | 75 |
