# Supplementary material for: Expected spatial patterns of alien woody plants in South Africa’s protected areas under current scenario of climate change
Source: Sci Rep. 2020 Apr 27;10:7038. doi: 10.1038/s41598-020-63830-x (PMC7184613; doi:10.1038/s41598-020-63830-x)
Supplement: Supplementary file 5 — Supplementary Table S4. [file 41598_2020_63830_MOESM5_ESM.docx]

**Expected spatial patterns of alien woody plants in South Africa’s protected areas under current scenario of climate change**

Bezeng S. Bezeng^1,2*^, Kowiyou Yessoufou^1^, Peter J. Taylor^2^, Solomon G. Tesfamichael^1^

*^1^Department of Geography, Environmental Management and Energy Studies, University of Johannesburg, APK Campus, Auckland Park 2006, South Africa.*

*^2^School of Mathematical & Natural Sciences, University of Venda, P. Bag X5050, Thohoyandou 0950, South Africa.*

Supplementary Table S4: Nineteen bioclimatic variables used as predictors in our SDMs.

| Abbreviation | Description |
| --- | --- |
| BIO1 | Annual Mean Temperature |
| BIO2 | Mean Diurnal Range (Mean of monthly max temp - min temp) |
| BIO3 | Isothermality (BIO2/BIO7) (* 100) |
| BIO4 | Temperature Seasonality (standard deviation *100) |
| BIO5 | Max Temperature of Warmest Month |
| BIO6 | Min Temperature of Coldest Month |
| BIO7 | Temperature Annual Range (BIO5-BIO6) |
| BIO8 | Mean Temperature of Wettest Quarter |
| BIO9 | Mean Temperature of Driest Quarter |
| BIO10 | Mean Temperature of Warmest Quarter |
| BIO11 | Mean Temperature of Coldest Quarter |
| BIO12 | Annual Precipitation |
| BIO13 | Precipitation of Wettest Month |
| BIO14 | Precipitation of Driest Month |
| BIO15 | Precipitation Seasonality (Coefficient of Variation) |
| BIO16 | Precipitation of Wettest Quarter |
| BIO17 | Precipitation of Driest Quarter |
| BIO18 | Precipitation of Warmest Quarter |
| BIO19 | Precipitation of Coldest Quarter |
