# Supplementary material for: Expected spatial patterns of alien woody plants in South Africa’s protected areas under current scenario of climate change
Source: Sci Rep. 2020 Apr 27;10:7038. doi: 10.1038/s41598-020-63830-x (PMC7184613; doi:10.1038/s41598-020-63830-x)
Supplement: Supplementary file 6 — Supplementary information. [file 41598_2020_63830_MOESM6_ESM.docx]

**Supplementary Materials:**

**SI Tables:**

Supplementary Table S1: Count of outliers within clusters of high or low invasion indices measured using Anselin Local Moran’s I z-score. HH = high value within a cluster of high values. HH = high value within a cluster of High values; HL = High within Low cluster; LH = Low within High cluster; LL = Low within Low cluster.

Supplementary Table S2: Plant invasion status of protected areas summarized by biome and classification types

Supplementary Table S3: Summary of invasion status indices of protected areas in South Africa

Supplementary Table S4: Nineteen bioclimatic variables used as predictors in our SDMs.

**SI Figures:**

Supplementary Figure S1: Spatial distribution of protected areas draped on biomes of South Africa. Map was prepared by the authors using ArcGIS software version 10.6.
